# Beyond biochemical patterning: How mechanical bistability governs robust organoid morphogenesis

**DOI:** 10.1016/j.mbm.2025.100134

**Published:** 2025-05-20

**Authors:** Qigan Gao, Yuehua Yang, Haoxiang Yang, Hongyuan Jiang

**Affiliations:** CAS Key Laboratory of Mechanical Behavior and Design of Materials, CAS Center for Excellence in Complex System Mechanics, Department of Modern Mechanics, University of Science and Technology of China, Hefei, Anhui, 230026, China

**Keywords:** Mechanical bistability, Intestinal organoids, Vertex model, Morphogenesis, Epithelial mechanics

## Abstract

Understanding the regulatory mechanisms of intestinal organoid morphogenesis remains a fundamental challenge in organoid biology. Emerging evidence highlights mechanical bistability as a critical regulator, mediated by dynamic lumen-actomyosin feedback. The recently developed 3D vertex model demonstrates that crypt curvature modulates actomyosin localization via mechanosensitive pathways, creating two stable morphological states—bulged or budded—depending on mechanical history. This model advances beyond static vertex models by incorporating epithelial thickness variations and lumen pressure effects, explaining previously unresolved phenomena like irreversible crypt budding and snap-through transitions. The findings establish a new framework for understanding mechanical decision-making in epithelial tissues, with implications for organoid engineering and developmental biology.

## Graphical abstract

**Figure:**
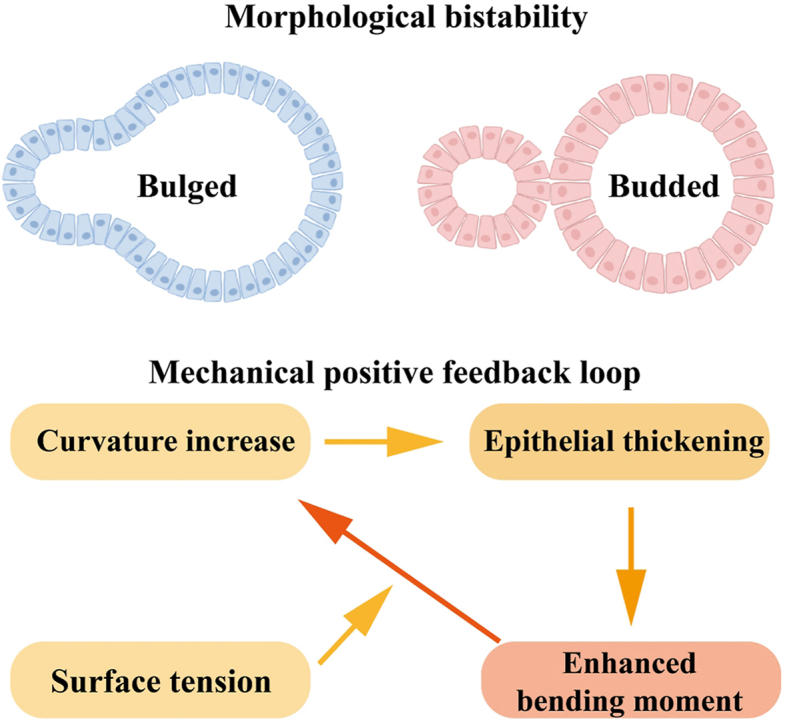

The process of morphogenesis—where cells self-organize into complex tissues and organs—has long fascinated biologists and physicists alike. Although biochemical signaling pathways have been extensively studied, the role of mechanical forces in shaping developing tissues has only recently gained prominence. Physical forces such as cytoskeletal tension, hydrostatic pressure, and tissue stiffness are now recognized as critical regulators of morphogenesis, working in concert with molecular cues to ensure robust pattern formation. Intestinal organoids, which recapitulate the crypt-villus architecture of the gut in vitro, have emerged as a powerful model system to study these mechanochemical interactions. However, a fundamental question remains: how do tissues maintain precise shapes despite inherent mechanical and biochemical noise? The study by Xue et al.^1^ addresses this gap by investigating the concept of mechanical bistability—a phenomenon where tissues can adopt one of two stable morphological states depending on their mechanical history. Their work not only advances our understanding of intestinal development but also provides a general framework for studying mechanical control in other organoid and developmental systems (see Fig. 1).

**Fig. 1:**
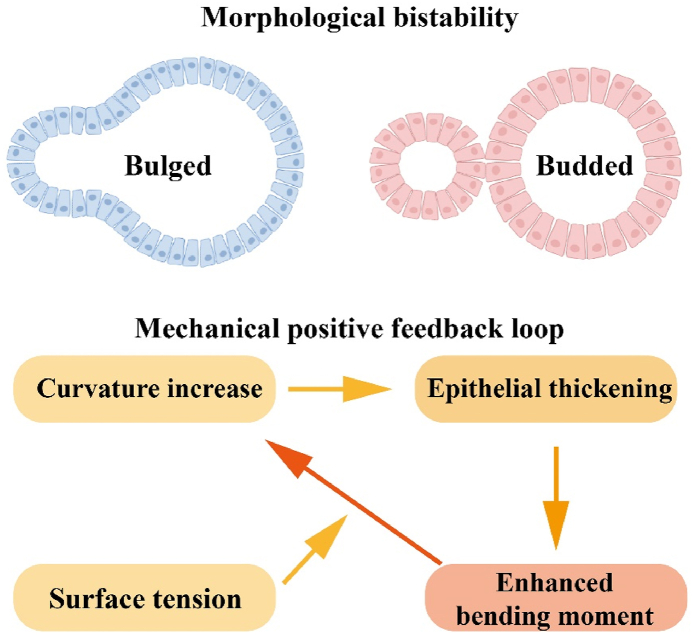
Schematic of the morphological bistability and mechanical positive feedback.

Recent studies found that volume regulation plays a critical role in modulating cell shape stability,^2^ cell migration,^3^ cell mechanical properties,^4^ and wave generation.^5^ The study by Xue et al.^1^ presents a novel 3D vertex model that provides unprecedented insight into how mechanochemical feedback between lumen volume changes and actomyosin tension governs intestinal organoid morphogenesis. Their model represents a significant theoretical advance over classical vertex approaches by incorporating three key innovations: dynamic tension regulation through mechanosensitive feedback, curvature-dependent epithelial thickness variations, and coupled lumen-epithelium mechanics. Unlike earlier vertex models that treated cellular tensions as static parameters,^6^^,^^7^ Xue et al.^1^ establish a dynamic feedback loop where crypt curvature actively modulates actomyosin localization through Piezo 1-mediated mechanotransduction pathways, creating a robust mechanical bistability between bulged and budded states. This represents a paradigm shift from previous organoid models like Serra et al.^8^ that attributed crypt formation solely to Wnt/Notch-mediated biochemical patterning while neglecting critical mechanical regulation.

The model's treatment of epithelial thickness variation represents a major theoretical breakthrough in vertex modeling approaches. In contrast to conventional vertex models that either assume constant cell height^9^ or implement passive height adjustments,^10^ Xue et al. demonstrate how active thickness-curvature coupling generates a mechanical positive feedback loop that stabilizes the budded state. This novel mechanism explains several previously puzzling observations in intestinal morphogenesis that were unaddressed by earlier folding models based on differential growth^11^ or passive buckling.^12^ Their integration of lumen pressure effects with active epithelial mechanics extends beyond classical epithelial vertex models, building upon recent advances in blastocyst lumen dynamics^13^ but introducing the crucial new dimension of mechanosensitive regulation through actomyosin redistribution.

When compared to other bistable systems in development, the Xue et al. model provides unique quantitative insights into mechanical hysteresis in epithelial tissues. Although bistability has been well-characterized in *Drosophila* mesoderm invagination^14^ and *Caenorhabditis elegans* embryonic polarization,^15^ these systems primarily involve biochemical feedback loops. The current work establishes a comprehensive framework for mechanical bistability in 3D epithelial structures, revealing how the energy barrier between states depends on lumen pressure and mechanosensation lowers this barrier to ensure robust transitions. The model's exceptional predictive power is demonstrated through its ability to recapitulate complex experimental observations that eluded previous intestinal organoid simulations,^16^ including the precise timing of irreversible crypt budding, snap-through transition dynamics with characteristic time scales of 20–30 minutes, and the maintenance of budded morphology despite subsequent mechanical perturbations. These capabilities establish the model as a new gold standard for studying mechanical decision-making in epithelial morphogenesis, with potential applications ranging from organoid engineering to understanding epithelial tumorigenesis.

The study opens several avenues for future research. First, the molecular mechanisms underlying curvature-sensing in crypt cells remain unclear. Identifying mechanosensors (e.g., Piezo 1 or YAP/TAZ) could deepen the understanding of feedback loops. Second, extending the model to in vivo contexts—where crypts interact with smooth muscle or stromal cells—would test its generalizability. Recent work^17^ shows substrate curvature biases crypt formation, suggesting external forces may modulate bistability. Third, integrating single-cell transcriptomics could reveal how mechanical states correlate with fate decisions. Finally, the framework could be adapted to other systems (e.g., brain organoids or tumor spheroids) where fluid pressure and tension interplay drives folding. Addressing these questions will require interdisciplinary efforts combining live imaging, optogenetics, and multiscale modeling.

## CRediT authorship contribution statement

**Qigan Gao:** Investigation, Writing – original draft. **Yuehua Yang:** Investigation, Writing – original draft. **Haoxiang Yang:** Investigation, Writing – original draft. **Hongyuan Jiang:** Conceptualization, Funding acquisition, Project administration, Supervision, Validation, Writing – review & editing.

## Ethical approval

This study does not contain any studies involving human or animal subjects.

## Declaration of competing interest

The authors declare that they have no known competing financial interests or personal relationships that could have appeared to influence the work reported in this paper.
